# Orthodontia

**Published:** 1901-07-15

**Authors:** C. Blair Blackmarr

**Affiliations:** Jackson, Mich.


THE
DENTAL REGISTER.

Vol. LV. July 15, 1901. No. 7.

### ORTHODONTIA.

BY C. BLAIR BLACKMARR, D.D.S., JACKSON, MICH.

Read before the Southwestern Michigan Dental Society, April 9, 1901.

Orthodontia should be one one of the first things thought of in the practice of dental surgery ; for surely after seeing that a child is well bred, well born and well nourished, the first thing thought of by a dental surgeon as he takes a baby for a patient is whether its teeth will come in and occlude normally and make a perfect temporary dental apparatus so as to properly masticate its food until such a time as the permanent set of teeth shall come to do the work. This child, from two to three years of age, is forming habits which may prevent a normal occlusion of the teeth. For instance, sucking the tongue, lip, thumb and rubber nipple, the results of which are very often unsightly.

An interesting case came to me for treatment lately. A child three years old was brought to me to see if anything could be done to its mouth to make it look normal. By watching the child a few moments only I saw that it sucked its lower lip most vigorously.

I asked the mother if the child was using a nursing bottle yet, and she said "yes." She was surprised to know, and you may be, that these two simple forces, *i.e.*, lip sucking and pressure of rubber nipple and bottle, would cause such a deformity, and it surely shows what little pressure it takes to make the teeth, and incidentally the jaws, take abnormal positions.

And now let us consider how much more liable a child's teeth are to become unclean and carious under such abnormal conditions than in a mouth in which there is normal occlusion. And from this age (three to six years) how important it is that the temporary teeth be kept in place normally, so that the first permanent molars take their places and occlude with each other in the normal way. How many children there are who have formed habits, or are forming habits, from the age of six to twelve years, which are causing their teeth to occlude abnormally: thumb sucking, mouth breathing, caused by nasal obstructions, etc.

Another case, which illustrates this same tendency, was caused by nasal obstructions, with enlarged tonsils and mouth breathing, causing an abnormal stretching force of the muscles of the mouth. The distance across the palate between the right and left second upper bicuspids is only a trifle over a quarter of an inch and less than three-eighths of an inch. These bicuspids are, however, crowded toward the palatal median line by the first molars, which have supplanted them and crowded them entirely out of the line of the arch.

Many children are victims of the bad habit ot extracting prematurely the temporary teeth, and especially the important first permanent molar; also the extracting of some of the temporory incisors or cuspids in the hope of giving room for erupting permanent incisors, and allowing the devitalized roots to remain in place beyond the normal period.

One writer has said: "It should be borne in mind that the interdependence of the teeth is so great at all times that the loss of one or more at any period in their history must have a marked influence upon the remaining teeth."

To illustrate the bad effects of premature extraction of the deciduous teeth, a case recently came into my hands for treatment that illustrates the statement in a forcible manner. In the upper jaw the permanent incisors, the permanent molars and the permanent first bicuspids are erupted and in the arch. The second temporary molars are the only deciduous teeth left in the arch and they occupy their proper positions. The temporary cuspids had been extracted, and the arch has contracted until it is closed, leaving no spaces for the permanent cuspids, which have not yet erupted but which can be distinctly seen appearing out of the line of occlusion and high up in the jaw under the lip. This condition was caused by the extraction of the temporary cuspids when the child was but eight years old, in order that the permanent lateral incisors might have room to get into the arch in proper alignment. The result was the contraction of the bones of the jaw and the closure of the arch, because the permanent cuspids had not sufficiently formed to come down into the space. As a natural consequence the jaw at that point has not developed sufficiently to admit the cuspids, which are detained in the jaw where developed. The dentist who extracted the cuspids tried to subvert nature's process by attempting to hold the space between the lateral and first bicuspid with a splint, but of course it could not be done. The contraction of the upper arch also produced contraction of the lower, and for some unwise reason the lower temporary molars have all been extracted, producing a recession of the permanent incisors and narrowing of the arch.

I have selected this case because it shows a not uncommon condition, a condition which should never occur at the hands of a dentist. It might happen from the care of some physicians who extract to relieve pain in children's teeth.

Now, in regard to the treatment of this case, I will say that I began it about February 1st by applying the "expansion arch" of the Angle system to the upper arch, allowing a week for the patient to become accustomed to its being in the mouth before allowing the arch to exert any force. March 1st I had sufficient space for the upper cuspids to erupt. The getting of these spaces in the two to three weeks' time was done with very little discomfort to the patient. I commenced operating on the lower arch March 25th; in a week this arch widened one-eighth of an inch, with no attempt to hurry it at all, the main purpose being to force out the cuspids and incisors.

Now there are some other points in this case that will be of interest to you—at least they were to me—in showing how easily the teeth and incidentally the alveolar process may be moved in any direction when taken at an early age. The older methods of "regulating teeth" so many times was to put some kind of a windlass appliance and try to bring in line some one offending tooth; but the cause or causes of its crooked condition were not taken into consideration.

From the studies of Prof. Angle there has been a great change. The importance of normal occlusion and the force of the occlusual planes, coupled with muscular force, harmony of the two arches and the classification into three distinct classes with their subdivisions, has simplified the whole matter and gives a more scientific basis to work upon. For each class there is of course a cause or causes which, if taken early enough, can all be avoided, and even if a little too late to avoid the results the effects can be remedied.

This boy is ten years of age, and by comparing measurements in his mouth and on models made at the time of commencing and on study models at the end of one month, it will be seen that the upper arch has been expanded three-eighths of an inch, measuring across from one first molar to the other on the upper jaw, and anteriorily a quarter of an inch. Again, the expression of the boy's mouth has changed from a contracted, pinched expression, not unlike a little old man's mouth, to a normal expression, round and full with pouting lips as a child's mouth should be.

The orthodox belief that doing right is a hard thing to do, and if done at all it must be done at a sacrifice to one's good feeling and happiness here on earth, but if we can stand it through we shall get our reward in heaven, has very likely done a great amount of good; but it seems to me it has likewise done a lot of harm by teaching people that some one else beside ourselves will save us from utter wreck and ruin. Some one else to pay our debts and suffer for our wrong doing has taught many a patient the idea that a dentist could save his teeth, no matter what he may not do. But I am glad to know that this old belief so long cherished as the *only* way is fast being discarded, more in accordance with justice and reason, and that it is doing right that comes out right, and doing wrong comes out wrong; that it is a pleasure in itself to do right and that the imagined pleasure of doing wrong is a delusion and a snare.

One very appreciative lady teacher in our public schools said to me upon my praising her for the excellent care she gave her teeth, "Why, I can not afford to do otherwise, although I should rather spend my money for something else rather than for having teeth filled." A very unappreciative person you may think, but very unlike the bright teacher, recently remarked in my hearing, "I have spent two hundred dollars having my teeth filled but it never did any good, for my teeth will not hold fillings." The disgusted person to whom he was making this remark replied, "If I had spent two hundred dollars to have my teeth filled I should spend fifty cents more for a tooth brush, and then I'd use it."

Prof. Moulton, of the University of Chicago, says: "In old Grecian times there were two distinct classes of people, one who read and the other who did not, and that to-day everybody reads; but the two classes are just as distinct as in the olden times, for one class thinks while it reads, the other class does not." And it seems to me this thinking class of to-day is realizing more and more that a normal dental apparatus occluding perfectly from youth to old age is possible, depending entirely upon the care given it by the individual owner, the same as with the eye, or any other organ in the human anatomy. This intelligent, appreciative class of people is very anxious to know why teeth decay, why irregular, why not straight, etc., and is more willing to pay a fee for advice in regard to the care of the dental apparatus than to pay for extracting, filling and laboratory work.

It is a matter of history, I believe, that the Empress of France in the reign of Napoleon had an aptness for hiding her defective teeth by the skillful manipulation of her ladyship's fan. Think of such an accomplishment for the first lady of the land to have to-day! Just imagine the number of people in the world to-day whose teeth have no care at all, either from themselves or dentists, and think of the amount of suffering this means; also think of the number of people who have extensive dental work done each year, all on account of the lack of knowledge in the care of their mouths. Of course, we dentists can not imagine the amount of suffering this last item means, because we are at the other end of the instruments. But patients could easily verify it.

What a contrast to these dark ages there would be if the dental profession would educate the people publicly and in private practice in the proper care of the teeth; and instead of using tons of "sen sen" and coffee to deodorize filthy mouths, and in place of picks, brushes and hot water, we should have clean, healthy mouths, with corresponding gums and teeth occluding normally. "The sweetest odor is no odor at all." Consider the greater satisfaction to a dentist having ten or even a hundred of these patients with modern ideas, instead of one of these "extensive job" patients. "The greatest good to the greatest number," you know.

Of course, at my age and with my disposition, you may imagine I am getting tired of filling teeth; but I believe if we should all do our best to teach people how to avoid having it done there would still be plenty of work to do as well as plenty of workers to do it. I really believe that if the blood supply in the jaw bone were properly directed, and the jaw properly exercised, we might often see such epitaphs as the following, which was taken, I believe, from a cemetery near Kalamazoo:

Here lies Brother Jonathan Gordon,
Mouth all mighty and teeth accordin'.
Stranger, step lightly o'er this wonder,
For if he opens his mouth, you 'r' a goner, by thunder.

The best recorded example of perfect manhood, so it is said, spent most of his time in relieving pain and teaching people how to avoid it, telling them to go and sin no more. We can not do better than to follow His example.

